# Choroid and choriocapillaris changes in early-stage Parkinson’s disease: a swept-source optical coherence tomography angiography-based cross-sectional study

**DOI:** 10.1186/s13195-023-01194-w

**Published:** 2023-02-20

**Authors:** Salih Uzun, Fatma Uzun

**Affiliations:** 1 Stanford University School of Medicine, Stanford, CA 94305 USA; 2 Ankara City Hospital, Ankara, Turkey

Dear Editor,

We have read and reviewed the article entitled *Choroid and choriocapillaris changes in early-stage Parkinson’s disease: a swept-source optical coherence tomography angiography-based cross-sectional study* by Zhang and colleagues with great interest [1]. The authors investigated the contrast sensitivity and performed swept-source optical coherence tomography (SS-OCT) and SS-OCTA to measure the outer retinal thickness, choroidal thickness (CT), choriocapillaris flow density, choroidal vascular volume (CVV), and choroidal vascular index (CVI).

The authors showed that there is a significant decrease in contrast sensitivity, outer retina thickness, choriocapillaris flow density, CVI, and CVV in patients with Parkinson’s disease. This research has also identified a positive correlation between outer retina thickness and contrast sensitivity. We congratulate the authors for their precious study, and we would like to request more details and their valuable contributions to the article.

The choroid is one of the most vascularized regions of the human body. Therefore, various local and systemic physiologic/pathologic conditions and environmental factors have effects on CT. It has been shown in the literature that age, sex, systemic/local diseases and their treatments, use of medicine, intraocular pressure, refractive error, axial length of the globe, and many other factors have effects on CT [2]. In addition, pregnancy, body mass index, menstrual cycle, and systemic blood pressure have remarkable effects on CT. Furthermore, consuming food and caffeinated or non-caffeinated drinks and exercising before OCT measurements can cause significant changes in CT [2]. Moreover, CT shows considerable diurnal variation and is able to increase by 50% in an hour and by four times in a few days [3]. We would like to ask the authors whether all those factors have been assessed in the study.

## Data Availability

Not applicable.
